# Supplementary material for: A Comprehensive Assessment of Ultraviolet-Radiation-Induced Mutations in Flammulina filiformis Using Whole-Genome Resequencing
Source: J Fungi (Basel). 2024 Mar 20;10(3):228. doi: 10.3390/jof10030228 (PMC10971301; doi:10.3390/jof10030228)
Supplement: Supplementary file 1 [file jof-10-00228-s001.zip › Supplementary Material S8/KEGG annotation/out/64381550635650.os/KO/out_map/map04740.html]

KEGG PATHWAY: Olfactory transduction - Reference pathway


|  |  |
| --- | --- |
| **Olfactory transduction - Reference pathway** |  |

[
Pathway menu
| Organism menu
| Pathway entry
| Show description
| User data mapping
]

Within the compact cilia of the olfactory receptor neurons (ORNs) a cascade of enzymatic activity transduces the binding of an odorant molecule to a receptor into an electrical signal that can be transmitted to the brain. Odorant molecules bind to a receptor protein (R) coupled to an olfactory specific Gs-protein (G) and activate a type III adenylyl cyclase (AC), increasing intracellular cAMP levels. cAMP targets an olfactory-specific cyclic-nucleotide gated ion channel (CNG), allowing cations, particularly Na and Ca, to flow down their electrochemical gradients into the cell, depolarizing the ORN. Furthermore, the Ca entering the cell is able to activate a Ca-activated Cl channel, which would allow Cl to flow out of the cell, thus further increasing the depolarization. Elevated intracellular Ca causes adaptation by at least two different molecular steps: inhibition of the activity of adenylyl cyclase via CAMKII-dependent phosphorylation and down-regulation of the affinity of the CNG channel to cAMP.

|  |  |
| --- | --- |
| Reference pathway | 100% |
